# Long-Term Outcomes of Behçet's Syndrome-Related Uveitis: A Monocentric Italian Experience

**DOI:** 10.1155/2020/6872402

**Published:** 2020-05-15

**Authors:** Jurgen Sota, Luca Cantarini, Antonio Vitale, Arianna Sgheri, Stefano Gentileschi, Valeria Caggiano, Viviana Gelardi, Bruno Frediani, Gian Marco Tosi, Claudia Fabiani

**Affiliations:** ^1^Research Center of Systemic Autoinflammatory Diseases and Behçet's Disease Clinic, Department of Medical Sciences, Surgery and Neurosciences, University of Siena, Siena, Italy; ^2^Ophthalmology Unit, Department of Medicine, Surgery and Neuroscience, University of Siena, Siena, Italy

## Abstract

**Objectives:**

To examine demographic and clinical characteristics and long-term visual outcome in a cohort of Italian patients affected by Behçet's uveitis (BU).

**Materials and Methods:**

Retrospective chart review of 47 patients with BU attending our unit between January 2018 and December 2019. Ophthalmologic manifestations, best-corrected visual acuity (BCVA), fluoroangiography and optical coherence tomography findings, and ocular complications were recorded. Predictive factors of a poor visual outcome and long-term complications were also investigated.

**Results:**

Forty-seven patients (23 males and 24 females) for a total of 84 eyes were enrolled. Uveitis was bilateral in 37 (78.7%) patients with panuveitis being the most frequent anatomical pattern (40 out of 84 eyes), whereas 27 eyes presented a posterior uveitis. Isolated anterior uveitis was detected in 16 eyes. A significant improvement of median BCVA between baseline and last follow-up values was detected (*p* = 0.042). A higher risk of poor visual prognosis was observed in patients with uveitis duration greater than 15 years (*p* = 0.019). A significant resolution of retinal vasculitis was detected between baseline and last follow-up evaluation (*p* < 0.0001) whereas the mean ± SD macular thickness did not decrease significantly between baseline (376.00 ± 97.45 *μ*m) and last follow-up evaluation (338.08 ± 55.81 *μ*m). Forty-two eyes developed 57 complications during the disease course. Cataract was the most frequent (*n* = 12), followed by epiretinal membranes (*n* = 11) and cystoid macular edema (*n* = 6). The following variables were identified as predictors of long-term complications: human leukocyte antigen- (HLA-) B51 (*p* = 0.006), panuveitis (*p* = 0.037), and a uveitis duration of more than 15 years (*p* = 0.049).

**Conclusions:**

In Italian patients, BU typically arises in the third decade and predominantly manifests as a bilateral posterior uveitis or panuveitis. Its duration is associated with a poor visual prognosis. Uveitis duration, the presence of HLA-B51, and panuveitis are predictors of long-term structural complications, thus representing main drivers in the treatment decision-making.

## 1. Introduction

Uveitis associated with Behçet's syndrome (BS) represents a severe intraocular inflammatory condition which plays a central role in the overall disease morbidity [1, 2]. The frequency of uveitis may vary according to several demographic variables. However, it is widely accepted that approximately half of BS patients have uveitis [2, 3]. Authors classically describe Behçet's uveitis (BU) as an acute, recurrent, nongranulomatous panuveitis associated with occlusive necrotizing retinal vasculitis compromising both arteries and veins [3, 4] and blindness occurring in a considerable proportion of patients [5–7]. Nevertheless, it is not rare to encounter an isolated involvement of the anterior segment and less often an intermediate uveitis [4, 8].

Severe uncontrolled uveitis relapses display a high risk of developing dreadful long-term complications, thus ending up in a poor visual prognosis. A substantial percentage of patients may experience a severe visual impairment [8] which is at least partially explained by the development of various irreversible structural complications such as optic atrophy, maculopathy, retinal atrophy, and neovascularization [4]. Additionally, uveitis may severely impair patients' quality of life going beyond the impact on visual acuity alone [9]. Posterior segment involvement has been associated with a poor visual prognosis and therefore is a major indication for an early and aggressive systemic treatment. More in detail, according to the EULAR recommendations, any BS patients with inflammatory disease affecting the posterior segment should be on systemic treatment with a conventional disease-modifying antirheumatic drug, interferon-alpha, or monoclonal antitumor necrosis factor (TNF) antibodies [10].

Solid epidemiologic data are a *sine qua non* condition for an accurate diagnostic work-up and for the establishment of an effective targeted therapy. BS is a relevant cause of a severe form of noninfectious uveitis in Italy, thus placing BS as a crucial element of the epidemiologic landscape of inflammatory ocular diseases in Italy [11].

Several studies have described BU in terms of clinical ocular features, complications, and potential risk factors predicting visual prognosis [6–8, 12–17]. We herein report our monocentric experience aimed at describing the uveitis characteristics and its clinical features focusing on long-term outcomes as well as finding predictors of visual outcomes and ocular complications.

## 2. Materials and Methods

### 2.1. Participants and Study Design

We conducted a retrospective analysis of medical records of a cohort of 47 patients affected by BU and admitted to the Rheumatology-Ophthalmology Collaborative Uveitis Center of the University Hospital of Siena between January 2018 and December 2019. BS diagnosis was based on the International Study Group (ISG) criteria [18] and/or International Criteria for Behçet's Disease (ICBD) [19]. Basic demographic and clinical information included gender, age, age at systemic disease onset, systemic disease duration, human leukocyte antigen- (HLA-) B51 typing, and clinical manifestations at the onset and throughout the disease course. Therapeutic data such as past and current treatments were also collected.

Patients undergoing biologic therapy were also screened by chest X-ray, QuantiFERON test, urine culture, and liver markers for HBV and HCV infections.

### 2.2. Ophthalmologic Examination and Study Objectives

Patients who had ocular involvement other than uveitis were excluded. A thorough ocular examination was performed in each patient. Best-corrected visual acuity (BCVA) was measured in a Snellen chart as a decimal fraction. Posterior segment involvement was assessed also through optical coherence tomography (OCT), OCT angiography, and/or fluorescein angiography (FA). Ophthalmological data collected also included age at uveitis onset, ocular complications, clinical activity, and the anatomical pattern established according to the SUN classification criteria [20]. Laterality was defined as either uni- or bilateral.

The primary aim of the study was to summarize ocular clinical features of BS patients followed up at our unit with particular focus on long-term outcomes and potential gender differences. Secondary aims consisted in detecting any possible impact of several variables on visual prognosis by identifying predictors of visual impairment and ocular complications.

### 2.3. Protocol Approval

The study adhered to the tenets of the declaration of Helsinki and received approval by the local Ethics Committee (Azienda Ospedaliera Universitaria Senese (AOUS); Reference No. 14951). Written informed consent was obtained from all patients or their legal guardians.

### 2.4. Statistical Analysis

Data were analyzed using IBM SPSS Statistics for Windows, version 24 (IBM Corp., Armonk, NY, United States). The normality of continuous data was assessed with the Shapiro-Wilk test. Descriptive statistics was used to calculate the mean and standard deviation (SD) or median and interquartile range (IQR) as appropriate. Categorical variables were analyzed by Pearson's chi-squared test or Fisher's exact test as required, while means were compared with Student's *t*-test for the independent variable test. McNemar and Wilcoxon tests were employed for categorical and continuous repeated measures, respectively. Variables including gender, HLA-B51 positivity, uveitis duration, retinal vasculitis, and panuveitis were inserted in the stepwise forward binary logistic regression to identify potential predictors of structural complications and poor visual outcome, the latter defined as a BCVA lower than 0.3 on a Snellen chart at the last follow-up. A *p* value of less than 0.05 was set as the threshold for statistical significance.

## 3. Results

### 3.1. Demographic Characteristics and Type of Uveitis

Forty-seven patients for a total of 84 eyes were enrolled in the study. Our cohort was composed of 24 female and 23 male patients with a male-to-female ratio of 0.96 : 1. Mean ± SD for age at uveitis onset was 33.81 ± 13.75 for the cohort as a whole, 31.78 ± 10.11 for males, and 35.75 ± 16.50 for females, without any statistical significance between genders (*p* = 0.324). Eight patients had a uveitis onset over 50 years of age. Demographic data alongside extraocular manifestations are shown in Table 1. Mean systemic disease duration at uveitis onset was 2.29 years.

Uveitis represented the first clinical manifestation of BS in 29 patients. It was bilateral in 37 patients and unilateral in 10 of them. Concerning the anatomical pattern, the majority of cases presented a posterior segment involvement with panuveitis being the most frequent (40 eyes) followed by posterior uveitis (27 eyes). In the remaining 17 eyes, 16 exhibited an isolated anterior uveitis and 1 eye had been enucleated before the admission in our unit for the severe anatomical consequences of a refractory panuveitis. In addition to uveitis, 2 eyes were concomitantly affected by scleritis (*n* = 1) and orbital inflammatory disease (*n* = 1).  Table 2 summarizes ocular features of our cohort including complications recorded at the last follow-up visit. No significant differences according to gender emerged among different variables taken into examination.

### 3.2. Visual Outcome

At the last follow-up, BCVA resulted greater than 0.5 in 69 eyes and equal or lower than 0.5 in 15 eyes, whereas 74 eyes had a visual acuity greater than 0.3. Three eyes were characterized by blindness at the last follow-up, with two of them already presenting with a BCVA equal to 0 at the baseline. A duration of uveitis of more than 10 and 15 years was significantly associated with a higher chance of having a BCVA of less or equal than 0.3 (*p* = 0.0413 and *p* = 0.0077, respectively). Regression analysis identified a higher risk of poor visual prognosis in patients with a uveitis duration greater than 15 years (OR 13.714 (C.I. 1.549-121.424), *p* = 0.019).

### 3.3. Complications

**Figure 1:**
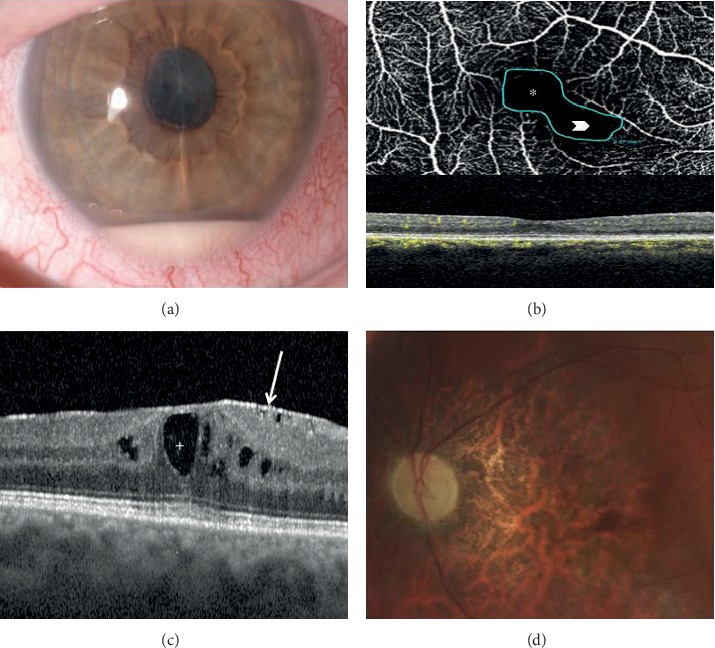
Sight-threatening complications of Behçet's syndrome- (BS-) related uveitis encountered in our cohort: (a) anterior chamber hypopyon in a case of BS-related panuveitis complicated with synechiae and cataract (right eye); (b) BS-related posterior uveitis complicated by macular epiretinal membrane (*arrow*) and chronic cystoid macular edema (*plus*) (right eye); (c) OCT angiography showing pathological enlargement of the foveal avascular zone (FAZ) (*asterisk*) along with a contiguous area of capillary dropout in the superficial retinal capillary plexus (*arrowhead*) as a sign of retinal ischemia (right eye); (d) optic nerve and macular atrophy due to the severe posterior pole vasculitis of the RE of a patient affected by refractory BS posterior uveitis.

Forty-two eyes developed 57 complications during the disease course. Cataract was the most frequently recorded complication (*n* = 12) followed by epiretinal membrane (*n* = 11) and cystoid macular edema (*n* = 6). Detailed information about ocular complications is provided in Table 2.  Figure 1 illustrates sight-threatening complications encountered in our cohort.

Several variables including HLA-B51 (*p* = 0.003), nonanterior uveitis (*p* = 0.021), and the presence of retinal vasculitis at baseline (*p* = 0.049), were associated with a significant higher rate of complications. When complications such as cataract and glaucoma were excluded, panuveitis also displayed a significant association with the development of structural complications (*p* = 0.0133). This association persisted also after adjusting for disease duration at regression analysis (OR 4.226 (1.182-15.112), *p* = 0.027). When excluding patients who presented only cataract and/or intraocular hypertension, at regression analysis, the following variables were identified as predictors of long-term complications: HLA-B51 (OR 9.156 (C.I. 1.871-44.806), *p* = 0.006), panuveitis (OR 4.601 (C.I. 1.094-19.348), *p* = 0.037), and a uveitis duration higher than 15 years (OR 4.605 (C.I. 1.002-21.168), *p* = 0.049).

Finally, a significant association was found between poor visual acuity and long-term complications (*p* = 0.048).

### 3.4. Treatment Impact

**Figure 2:**
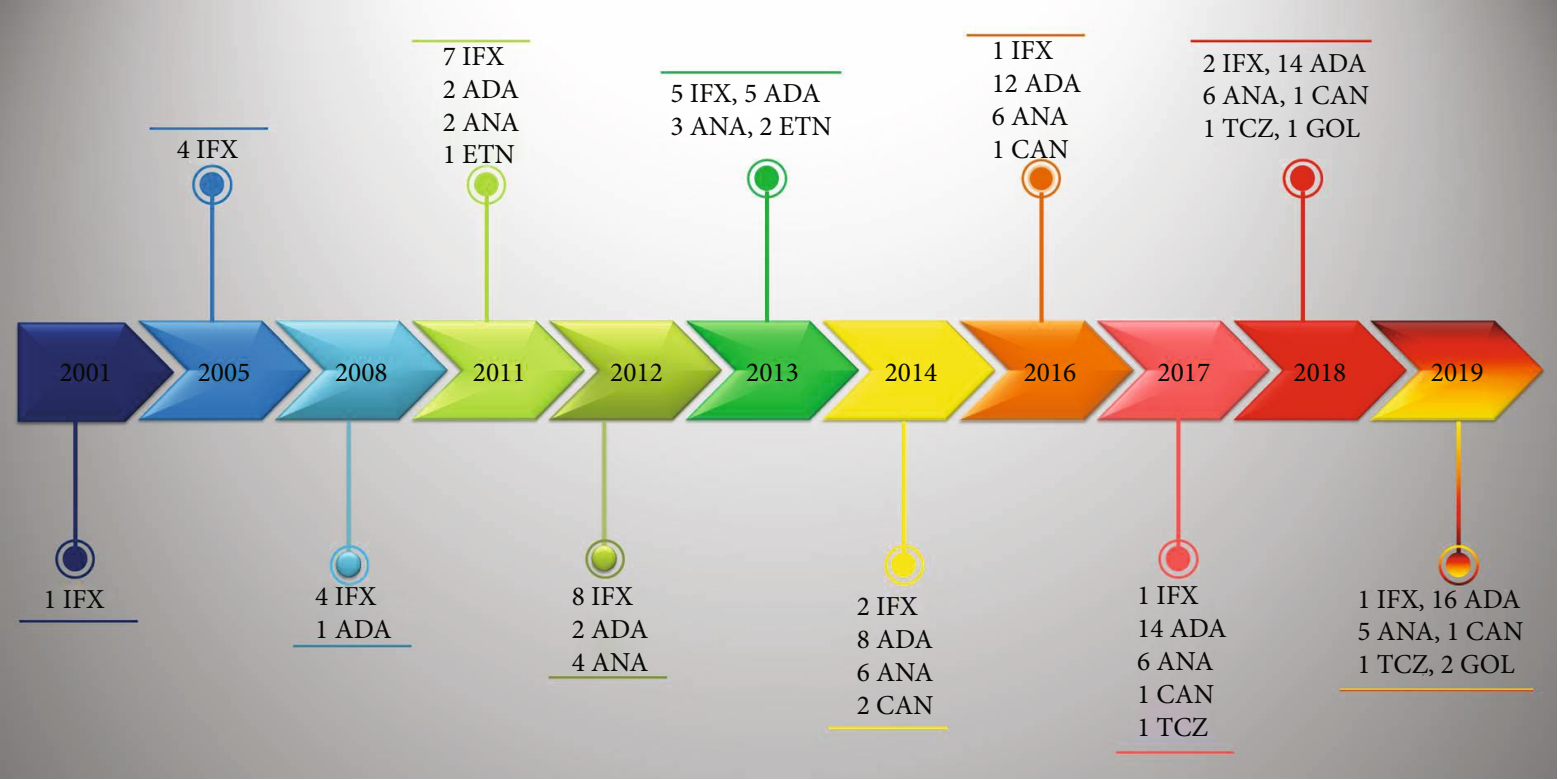
Timeline graph illustrating the different biologic treatments administered to our patients over time. List of abbreviations: ADA: adalimumab; ANA: anakinra; CAN: canakinumab; ETN: etanercept; GOL: golimumab; IFX: infliximab; TCZ: tocilizumab.

With regard to treatment modalities, 27 patients received only 1 biologic agent during the follow-up, 15 were treated with 2 different biologics at different time points, and 8 of them had been treated with 3 or more different biologics. No significant differences in terms of development of structural complications emerged between patients treated with biologics and those receiving conventional immunosuppressive treatment (*p* = 0.90).  Figure 2 shows a timeline graph of different biologic treatments received during the follow-up, whereas preceding and current treatments are displayed in Table 3.

Median ± IQR BCVA at baseline and last follow-up evaluation was equal to 1.0 ± 0.38 and 1.0 ± 0.18, respectively, and the difference between these two timepoints was statistically significant (*p* = 0.042).

**Figure 3:**
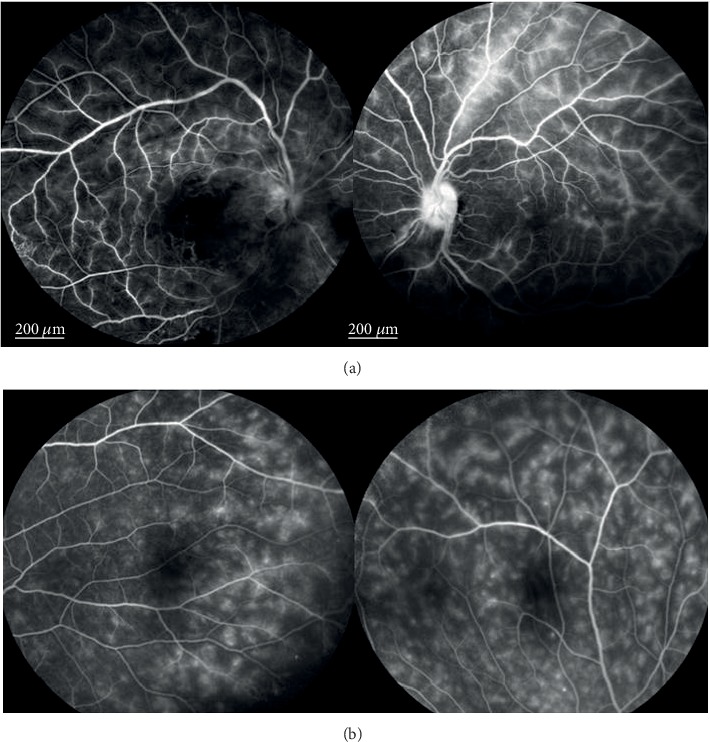
Fluorescein angiography (FA) findings in a patient with severe retinal vasculitis; (a) late-phase FA showing optic disc and vascular leakage and areas of nonperfusion in a Behçet's syndrome (BS) patient with a bilateral severe occlusive vasculitis; (b) late-phase FA in a patient with active bilateral retinal vasculitis affected by BS. All retinal peripheral quadrants of both eyes of this patient showed the same diffuse fern-like capillary leakage, the most frequent FA finding for Behçet's uveitis.

A significant resolution of retinal vasculitis was detected between baseline and last follow-up evaluation (*p* < 0.0001). At baseline, 28 posterior pole retinal vasculitides were identified on FA, and 16 of them also involved the optic disk.  Figure 3 displays FA findings of a case with severe retinal vasculitis involving both posterior pole (Figure 3(a)) and peripheral retinal quadrants (Figure 3(b)).

OCT findings improved from 376.00 ± 97.45 *μ*m to 338.08 ± 55.81 *μ*m, without any significance between baseline values and the last follow-up examination.

## 4. Discussion

Uveitis represents by far the most frequent and typical ocular manifestation of BS. BU and particularly posterior segment inflammatory involvement may cause severe and irreversible damage of the optic nerve and the retinal structures causing permanent visual loss and even blindness [4]. Patients with BS-related panuveitis and visual impairment present a poorer quality of life compared to healthy controls and to BS patients without ocular involvement [21]. In our series, panuveitis and posterior uveitis taken together accounted for almost 80% of the sample, constituting approximately a similar proportion reported in previous studies [6, 7, 14–16]. Uveitis represented the first clinical manifestation in 29 patients. Interestingly, among this subgroup of patients, 12 of them were free of other suggestive signs of BS at the uveitis onset. This finding advocates for a close follow-up of any idiopathic nonanterior uveitis, especially when typical ophthalmological BS-uveitis findings are present [4]. Mean age at onset was similar to previous reports ranging from late 20s to early 30s [6, 8, 13–15].

No differences between male and female patients regarding age at uveitis onset, anatomical pattern, visual prognosis, and the development of long-term structural complications emerged. The lack of gender differences may be partially explained by the relatively small sample size. Indeed, our study group has recently published a large epidemiologic study where male patients were found to be significantly more affected by posterior uveitis and panuveitis [22]. Another possible explanation may reside in the prognostic evolution of BS in recent decades. More specifically, the risk of losing useful vision has been significantly reduced over time and the BU prognosis is expected to improve in the near future also in the light of the availability of more targeted therapies [8, 23, 24]. Moreover, BS patients with poor vision in Italy have been found to be significantly fewer than those in endemic areas [15].

Innovative therapies account for a major contribution in the prognostic evolution of BU. Indeed, the advent of biologic agents has considerably revolutionized its management. In particular, anti-TNF-*α* monoclonal antibodies elicit a highly suppressive effect on ocular inflammatory attacks and have proven to be effective in the treatment of uveitis related to BS by improving visual acuity, showing a good long-term drug retention rate and a steroid-sparing effect [25–28]. In refractory cases, interleukin-1 inhibition may represent a valid alternative therapeutic approach [29]. In the present study, we observed a significant improvement of the median BCVA values and a decrease in the frequency of retinal vasculitis between baseline and last follow-up examination. The lack of significant decrease of the mean OCT values may be explained by the development of macular complications such as epiretinal membranes in a large proportion of patients during the disease course. Despite the ability to preserve visual function and reduce the relapses of retinal vasculitis, treatment with biologic agents was not associated with a reduced rate of long-term structural complications when compared with patients receiving conventional immunosuppressive drugs. It is possible that the lack of a significant decrease in OCT values and the already high mean disease duration at baseline may account for their inability to reduce long-term structural complications. An increased awareness along with an early diagnosis and a prompt introduction of systemic immunosuppressive therapy may halt the natural disease course, thus lowering the rate of long-term complications and poor visual acuity.

In our cohort 42 out of 84 eyes (50%) developed at least one complication with the most frequent being cataract, epiretinal membranes, and cystoid macular edema. Several variables were found to predict their long-term occurrence. A German study group identified a higher frequency of HLA-B51 among patients developing ocular involvement [16]. With regard to its association with ocular involvement and type of uveitis, current medical literature displays controversial results [30, 31]. However, several authors consider HLA-B51 as a marker of unfavorable prognosis [32, 33]. In line with the latter statement, our findings highlight the role of HLA-B51 as a predictor of long-term structural complications and subsequently of poor visual outcome. As expected, also posterior segment involvement and a long uveitis duration were identified as further predictors of long-term complications.

Low visual acuity at presentation and the development of a severe complication such as optic disc atrophy have been linked to a poorer visual outcome [6]. We could not evaluate these variables due to a low number of optic atrophy cases and a substantially good visual acuity at baseline evaluation. Despite its vital importance, the identification of predictive factors of poor long-term prognosis in BS is currently an understudied aspect. A deeper knowledge of the variables conferring a worse visual prognosis may allow a better patient profiling and therefore orientate a more precise and targeted therapy. Based on our findings, we suggest that HLA-B51 positivity, posterior segment involvement, and longer uveitis duration are strong drivers in the therapeutic decision. These findings however deserve further investigation and should be interpreted in the context of several limitations such as the retrospective design accompanied by its inherited shortcomings. Indeed, some interesting variables such as comorbidities, which may behave as confounding factors, were not collected for the purposes of this study. The relatively small sample size may also be responsible for the wide confidence intervals detected in regression analysis, which do not provide a precise magnitude of effect, highlighting the need for further studies with larger sample sizes. Additionally, the referral bias due to the tertiary referral nature of our center might exclude milder forms of intraocular inflammation and hence be only representative of the complicated end of disease spectrum.

In conclusion, according to our experience, BU arises in the third decade of life and predominantly compromises the posterior chamber as posterior uveitis or panuveitis, without any gender influence. Its duration seems to be associated with a poor visual acuity. Uveitis duration along with the presence of HLA-B51 and panuveitis predict the development of long-term structural complications, thus representing main drivers in the treatment decision-making. Despite the therapeutic advances and an improved prognosis compared to the past decades, uveitis associated with BS remains a challenging and hard-to-handle condition. A better understanding of its epidemiologic landscape is essential to prevent or at least minimize irreversible and structural damage through patient profiling with the aim to identify high-risk subjects.

## Figures and Tables

**Table 1:** Demographic and extraocular features of our cohort at disease onset and throughout the course of Behçet's syndrome.

Demographic features	Mean ± SD expressed in years
Female/male	23/24
Age	48.68 ± 14.38
Age at BS onset	31.17 ± 13.37
Age at uveitis onset	33.81 ± 13.75
Uveitis duration	15.21 ± 8.82
HLA-B51	27/47 (57.45%)
*Clinical features at onset*	*N(%)*
Uveitis	31 (65.96%)
Oral aphthosis	34 (72.34%)
Genital aphthosis	9 (19.15%)
Erythema nodosum	2 (4.25%)
Pseudofollicular lesions	7 (14.89%)
CNS involvement	2 (4.25%)
Gastrointestinal involvement	4 (8.51%)
Vascular involvement	2 (4.25%)
*Clinical features throughout BS course*	*N(%)*
Oral aphthosis	46 (97.87%)
Genital aphthosis	19 (40.43%)
Erythema nodosum	8 (17.02%)
Pseudofollicular lesions	24 (51.06%)
CNS involvement	8 (17.02%)
Gastrointestinal involvement	15 (31.91%)
Vascular involvement	12 (25.53%)

List of abbreviations: BS: Behçet's syndrome; CNS: central nervous system; HLA: human leukocyte antigen; IQR: interquartile range; SD: standard deviation.

**Table 2:** Ocular characteristics and complications in Behçet's uveitis in Italian patients.

Characteristics	Number of eyes (%)
*Anatomical location*	
(i) Anterior uveitis	16
(ii) Posterior uveitis	27
(iii) Panuveitis	40
*Retinal vasculitis at baseline*	
(i) Posterior pole vasculitis	28
(ii) Optic disk vasculitis	16
(iii) Peripheral vasculitis	7
*Complications*	
Cataract	12
Maculopathy	
(i) Epiretinal membranes	11
(ii) Macular edema	6
(iii) Macular atrophy	5
(iv) Macular hole	1
Glaucoma	5
Ocular hypertension (IOP > 25 mmHg)	2
Others	
(i) Synechiae	6
(ii) Peripheral retinal ischemia	4
(iii) Optic disc atrophy	2
(iv) Retinal detachment	1
(v) Optic disc neovascularization	1
(vi) Phthisis bulbi	1

List of abbreviations: IOP: intraocular pressure.

**Table 3:** Past and current treatment in our cohort.

*Previous cDMARDs*		*Current cDMARDs*	
CSA	16	CSA	5
AZA	12	AZA	5
MTX	14	MTX	3
SZS	4	SZS	1
LFN	1	HCQ	1
CYC	1		
Thalidomide	1		

*Previous biologics*		*Current biologics*	
ADA	18	ADA	11
IFX	10	IFX	2
GOL	2	GOL	1
ANA	9	ANA	4
CAN	1	CAN	2
ETN	4	TCZ	1
TCZ	1		

List of abbreviations: ADA: adalimumab; ANA: anakinra; AZA: azathioprine; CAN: canakinumab; cDMARDs: conventional disease-modifying antirheumatic drugs; Cyc A: cyclosporin A; CYC: cyclophosphamide; ETN: etanercept; GOL: golimumab; HCQ: hydroxychloroquine; IFX: infliximab; LFN: leflunomide; MTX: methotrexate; SZS: sulfasalazine; TCZ: tocilizumab.

## Data Availability

The datasets generated for this study are available on request to the corresponding author.
